# The Impact of Electronic Medical Records on Healthcare Professionals’ Satisfaction and Service Delivery at the Federal Medical Centre, Gusau

**DOI:** 10.7759/cureus.95761

**Published:** 2025-10-30

**Authors:** Usman Hosea Ojoh, Simeon Suurshater Tughemba, Usman Fatima

**Affiliations:** 1 Department of Oncology, Federal Medical Centre, Gusau, NGA; 2 Department of Medicine and Surgery, Ahmadu Bello University Teaching Hospital, Zaria, NGA; 3 Department of Family Medicine, Kano State Primary Healthcare, Kano, NGA

**Keywords:** electronic medical records, healthcare delivery, nigeria, satisfaction, workflow

## Abstract

Background

Electronic medical records (EMRs) are vital tools in enhancing healthcare delivery through better documentation, communication, and data retrieval. In low- and middle-income countries (LMICs), including Nigeria, EMR implementation faces challenges such as limited infrastructure, poor usability, and insufficient training. This study assesses the impact of EMRs on healthcare professionals' satisfaction and its effect on healthcare service delivery at Federal Medical Centre (FMC) Gusau, identifying key predictors and challenges to inform implementation strategies.

Methods

A cross-sectional study was conducted at FMC Gusau, Zamfara State, Nigeria, a tertiary healthcare facility, from October 2024 to January 2025. Among 415 clinical staff selected through stratified random sampling after data cleaning, responses with more than 50% unfilled fields were removed from the initial 423; a semi-structured questionnaire was adapted, modified, and pretested for internal consistency (Cronbach alpha 0.88 with 10% sample, n=42) to evaluate EMR satisfaction, service delivery impact, and utilization challenges. Data were analyzed using SPSS version 20 (IBM Inc., Armonk, New York); summary statistics included frequencies and percentages for categorical variables, means for normally distributed continuous variables, and medians with interquartile ranges for skewed data. Ordinal logistic regression was used to identify predictors of satisfaction (p<0.05).

Results

Of the 415 respondents, 36 (8.7%) reported high satisfaction, 192 (46.3%) reported moderate satisfaction, and 187 (45.1%) reported dissatisfaction. Satisfaction was highest for system navigability, 294 (70.9%), and data retrieval speed, 303 (73.0%). EMRs were perceived to improve documentation 279 (67.2%), record access 334 (80.4%), and interdepartmental communication 265 (63.9%), though 204(49.2%) noted increased documentation time. Training (AOR=2.68, 95% CI=1.52-4.71, p=0.001) was a significant predictor, while duration of practice showed no significant effect (AOR=0.95, 95% CI=0.55-1.64, p=0.852). Major challenges included limited training 108 (26.0%), IT support 97 (23.3%), and interface issues 80 (19.3%).

Conclusion

EMRs offer clear benefits in improving healthcare delivery, yet user satisfaction remains moderate due to technical and training-related barriers. Strengthening training, IT support, and user-centered design is crucial for effective EMR adoption and sustainability in resource-constrained settings.

## Introduction

Electronic medical records (EMRs) are increasingly recognized as essential tools for improving healthcare delivery through enhanced documentation, accessibility, and inter-professional communication [1,2]. In low- and middle-income countries (LMICs), where limited resources often strain healthcare systems, EMRs offer opportunities to bridge gaps in care quality, efficiency, and data-driven decision making [3,4]. Despite these benefits, EMR implementation in LMICs has been met with challenges, including limited technical infrastructure, insufficient user training, and resistance to change [3,5,6]. These challenges impact healthcare providers' satisfaction and the effectiveness of EMRs in clinical settings. Satisfaction, a key determinant of system usability and sustainability, is impacted by factors such as ease of navigation, timeliness of data retrieval, and perceived workload reduction [6,7].

Healthcare professionals in LMICs often report dissatisfaction with EMR systems due to usability issues, poor interface design, and frequent technical downtimes, leading to decreased efficiency and workflow disruptions [4-6]. These problems hinder the potential of EMRs to support quality healthcare delivery.

While numerous studies have evaluated EMR satisfaction in high-income countries, there is limited evidence from LMICs, particularly in Nigeria. This study aims to assess healthcare professionals’ satisfaction with EMRs and the impact of EMRs on service delivery in a tertiary hospital setting, identifying key predictors and challenges to inform implementation strategies.

## Materials and methods

Study area

Federal Medical Centre (FMC) Gusau, located in Zamfara State, Nigeria, is a tertiary healthcare facility serving as a referral center for primary and secondary facilities across Zamfara and neighboring regions. FMC Gusau uses a standard digital EMR system for patient records, implemented over a year before this study, with varying adoption across departments, providing a relevant context for assessing EMR satisfaction and challenges.

Study population

The study population was the clinical staff at FMC Gusau during the study period (October 2024 to January 2025).

Inclusion criteria

Doctors, nurses, pharmacists, medical laboratory scientists, physiotherapists, nutritionists, and other clinical staff who use the hospital's EMR system were included in the study

Study design

Cross-sectional design to assess EMR satisfaction, impact on workflow efficiency, resource allocation, and challenges.

Sample size determination

Sample size was calculated using the standard formula for cross-sectional studies [8]: N=Z²pq/d² where Z=1.96, p=0.53 [9], q=1-p, and d=0.05. This yielded 385, and with a 10% non-response adjustment, the initial sample was 423. After data cleaning (removing responses <50% filled), 415 responses remained.

Sampling technique

Stratified sampling by category, with proportionate allocation and random selection using the WINPEPI software. Informed consent was obtained; non-consenting individuals were excluded.

Instrument for data collection 

The questionnaire for this study was developed by adapting validated items from prior studies on electronic medical record (EMR) implementation and healthcare professional perspectives, including Senishaw et al. [10] and Alobo et al. [11], which explored willingness to adopt EMRs and implementation challenges in similar contexts. These studies informed the design of sections on satisfaction, workflow impact, and utilization barriers, tailored to the local setting at Federal Medical Centre Gusau. The instrument was further modified and pretested for internal consistency (Cronbach alpha 0.88 with a 10% sample, n=42) to evaluate EMR satisfaction, service delivery impact, and utilization challenges. Scoring was conducted by aggregating responses on a 5-point Likert scale (1 = strongly disagree to 5 = strongly agree), with satisfaction categorized as poor (scores 1-2), moderate (score 3), and high (scores 4-5) for ordinal logistic regression analysis.

Data analysis

Data was analyzed using SPSS version 20 (IBM Inc., Armonk, New York). Ordinal logistic regression identified satisfaction predictors (p<0.05). Summary statistics, including frequencies, percentages, and means, were employed as detailed in the results section.

## Results

Analysis of 415 respondents at the Federal Medical Centre, Gusau, revealed varied levels of satisfaction and the impact of service delivery associated with the electronic medical records (EMR) system. Findings are detailed across socio-demographic profiles, satisfaction metrics, predictive factors, and workflow/resource outcomes.

Table 1 outlines the socio-demographic characteristics of the 415 healthcare professionals, revealing a young workforce with a mean age of 31.9 ± 6.6 years and 77.8% aged 35 or below. The sample was predominantly female (57.8%), with a median duration of practice of three years (IQR: 2-5), nurses forming the largest group (52%), and 63.1% having received EMR training.

**Table 1:** The socio-demographic characteristics of the respondents SD - standard deviation; IQR - interquartile range; EMR - electronic medical record

Characteristics	Value, n (%)
Age group (years)	
≤35	323 (77.8)
36 and above	92 (22.2)
Mean±SD	31.9±6.6 years
Sex	
Male	175 (42.2)
Female	240 (57.8)
Duration of practice(years)	
≤5	317 (76.4)
6 and above	98 (23.6)
Median(IQR)	3 (2-5)
Category of healthcare professionals	
Medical doctors	133 (32)
Nurses	216 (52)
Pharmacist	22 (5.3)
Others	44 (10.6)
Training on EMR	
Yes	262 (63.1)
No	153 (36.9)

Table 2 presents satisfaction levels with the EMR system, differentiating individual performance metrics from overall satisfaction. For individual aspects, 70.9% rated navigability as easy or very easy, and 73.0% found data retrieval timeliness satisfactory or very satisfactory. Overall satisfaction, evaluated independently, indicated 8.7% were highly satisfied, 46.3% moderately satisfied, and 45.1% dissatisfied, providing a comprehensive view of the varied responses to specific features versus general system perception.

**Table 2:** Satisfaction of healthcare professionals with electronic medical record system EMR - electronic medical record

Characteristics	Value, n (%)
Ease of navigating EMRs	
Very easy	156 (37.6)
Easy	138 (33.3)
Undecided	61 (14.7)
Difficult	49 (11.8)
Very difficult	11 (2.7)
User friendliness with EMRs	
Very satisfied	157 (37.8)
Satisfied	136 (32.8)
Undecided	86 (20.7)
Dissatisfied	34 (8.2)
Very dissatisfied	2 (0.5)
Timeliness of retrieval of information	
Very satisfied	149 (35.9)
Satisfied	154 (37.1)
Undecided	80 (19.3)
Dissatisfied	30 (7.2)
Very dissatisfied	2 (0.5)
Frequency of technical issues with EMRs	
Never	16 (3.9)
Rarely	74 (17.8)
Occasionally	169 (40.7)
Frequently	135 (32.5)
Always	21 (5.1)
Overall satisfaction	
Excellent	90 (21.7)
Good	213 (51.3)
Fair	81 (19.5)
Poor	20 (4.8)
Very poor	11 (2.7)
Satisfaction level	
Unsatisfied	187 (45.1)
Moderately	192 (46.3)
Satisfied	36 (8.7)

Table 3 details logistic regression predictors of satisfaction, highlighting training as a significant factor (adjusted odds ratio (AOR) = 2.68, 95% confidence interval (CI)=1.52-4.71, p=0.001), while age, sex, duration of practice, and professional category showed no significant effects (e.g., duration AOR = 0.95, 95% CI=0.55-1.64, p=0.852).

**Table 3:** Ordinal logistic prediction of satisfaction with electronic medical record AOR - adjusted odds ratio; EMR - electronic medical record

Factors	AOR	95% CI	-value
Age group (years)			
<35	1.3	0.4-1.434	0.445
>35	1	-	-
Sex			
Female	1.32	0.80-2.18	0.272
Male	1	-	-
Duration of practice (years)			
≤5	0.952	0.55-1.64	0.852
>5	1	-	-
Training on EMR			
Yes	2.68	1.52-4.71	0.001
No	1	-	-
Categories of healthcare professionals			
Medical doctors	0.71	0.27-1.186	0.489
Nurses	0.43	0.17-1.08	0.073
Pharmacist	1	-	-
Others	0.42	0.14-1.22	0.109

Table 4 examines the impact of EMRs on clinical workflow efficiency, as 67.2% reported an improvement in documentation efficiency, with 67.2% reporting improvements in documentation (rated as somewhat to very well), and 80.4% found record retrieval highly accessible (rated as easy to very easy). However, 49.2% noted an increase in documentation time, while 63.9% acknowledged a significant enhancement in interdepartmental communication.

**Table 4:** Impact of EMRs on clinical workflow efficiency EMR - electronic medical record

Characteristics	Value, n (%)
Streamline documentation process	
Very poorly	7 (1.7)
Poorly	25 (6)
Undecided	104 (25.1)
Somewhat well	122 (29.4)
Very well	157 (37.8)
Ease of retrieval of patient record	
Difficult	17 (4.1)
Undecided	64 (15.4)
Easy	145 (34.9)
Very easy	189 (45.5)
Impact on time spent on patient documentation	
Significantly increased	107 (25.8)
Slightly increased	97 (23.4)
Undecided	88 (21.2)
Slightly reduced	80 (19.3)
Significantly reduced	43 (10.4)
Improvement in interdepartmental communication	
Very poor	14 (3.4)
Poor	35 (8.4)
Undecided	101 (24.3)
Somewhat well	160 (38.6)
Very well	105 (25.3)
Reduce delay in accessing patient information	
Very poor	5 (1.2)
Poor	17 (4.1)
Undecided	60 (14.5)
Somewhat well	163 (39.3)
Very well	170 (41.0)

Table 5 presents data indicating 66.0% optimization of hospital resource utilization (rated as somewhat to very well), 66.3% effectiveness in workload management, and 75.4% confidence in enhancing technology investments, alongside an overall positive impact rated as good to excellent by 67.7% of respondents.

**Table 5:** Impact of EMRs on resource allocation EMR - electronic medical record

Characteristics	Value, n (%)
How EMRs optimize resources within hospital	
Very poor	22 (5.3)
Poor	30 (7.2)
Undecided	89 (21.4)
Somewhat well	135 (32.5)
Very well	139 (33.5)
Effectiveness in managing staff workload	
Very poor	12 (2.9)
Poor	50 (12.0)
Undecided	78 (18.8)
Somewhat effective	144 (34.7)
Very effective	131 (31.6)
Support financial resource tracking	
Very poor	6 (1.4)
Poor	40 (9.6)
Undecided	60 (14.5)
Somewhat well	145 (34.9)
Very well	164 (39.5)
Confidence in EMRs to enhance technology investment	
Not confident at all	5 (1.2)
Somewhat not confident	21 (5.1)
Undecided	76 (18.3)
Somewhat confident	130 (31.3)
Very confident	183 (44.1)
Overall impact on hospital resource allocation	
Very poor	4 (1.0)
Poor	27 (6.5)
Fair	103 (24.8)
Good	164 (39.5)
Excellent	117 (28.2)

**Figure 1:**
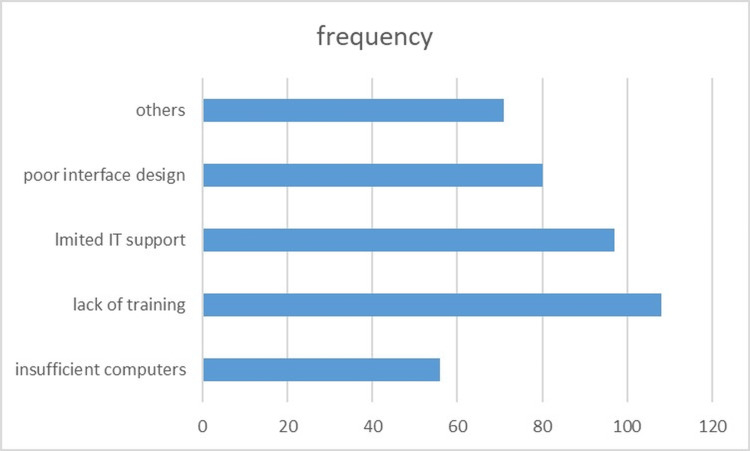
Challenges with EMR use among healthcare workers at the Federal Medical Centre, Gusau EMR = electronic medical record

Figure 1 depicts the distribution of EMR challenges among the 415 respondents, with lack of training identified as the most prevalent issue (26%, n=109), followed by limited IT support (24%, n=98), poor interface design (19%, n=80), other issues (17%, n=72), and insufficient access to computers (14%, n=56)

## Discussion

Electronic medical records (EMRs) play a critical role in improving the quality and efficiency of healthcare delivery, particularly in resource-constrained settings. Their implementation facilitates accurate documentation, faster access to patient records, improved interdepartmental coordination, and better resource utilization. In low- and middle-income countries (LMICs) such as Nigeria, EMR adoption is increasingly regarded as a strategic approach to modernizing health systems and addressing inefficiencies inherent in paper-based documentation [2-4]. This study evaluated the impact of electronic medical records (EMRs) on healthcare professionals' satisfaction and service delivery at Federal Medical Centre, Gusau, Nigeria, using a cross-sectional design involving 415 clinical staff. The moderate satisfaction levels observed (8.7% high, 46.3% moderate) are comparable to regional studies in LMICs, particularly Ethiopia. For instance, a cross-sectional study in Ethiopia reported 53.1% satisfaction among healthcare professionals, with training and usability as key factors [8], suggesting a slightly higher acceptance rate than in Gusau. Willingness to adopt EMRs reached 75.6% in Ethiopia's Amhara region with adequate training [10], indicating that the 26% training barrier in this study (n=108) could be a critical intervention point. Positive attitudes (58.3%) among Ethiopian physicians were linked to computer literacy [12, 13], while in Malaysia, nurses reported 70.9% moderate satisfaction, driven by technical support [9], aligning with the 70.9% navigability satisfaction but contrasting with the overall dissatisfaction trend.

In Nigeria, a specialist hospital study found 45.5% satisfaction, with similar challenges like power outages [11], mirroring the 45.1% dissatisfaction rate. A multi-state Nigerian study noted 70-95% positive perceptions tied to usefulness [12], suggesting Gusau's 67.2% documentation improvement lags behind potential. Rwandan healthcare workers reported an over 90% agreement on the benefits of EHRs for decision-making [14]. A recent Nigerian review highlighted the impact of EMR on delivery, supporting improvements of 67.2-80.4% in documentation and access [15], which exceeded the 80.4% satisfaction rate for record access. These comparisons highlight that while EMRs improve efficiency in LMICs, satisfaction varies with local implementation quality [16]. Training represents the most significant gap and strongest predictor of EMR satisfaction, with 49.2% of staff citing lack of training (Figure 1) despite 65.1% coverage (Table 1). The AOR=2.68 (p=0.001) (Table 3) shows trained staff are 2.68 times more likely to report high satisfaction (73.0%), establishing training as the priority intervention for EMR success at FMC, Gusau. Findings in this study further support LMIC evidence where training on EMR increased satisfaction odds by 4.00 in Ethiopia [8] and 3.29 in Amhara [10], and underscore the critical role of training in EMR implementations [5,6]. The negative association with duration of practice (AOR=0.952, p=0.852) may reflect resistance among experienced staff, differing from studies where longer experience enhanced adoption [15]. The 67.2% documentation improvement and 80.4% record access rate affirm EMRs' potential to bridge care gaps [3,4], but the 49.2% increase in documentation time aligns with global usability concerns [4-6]. Challenges like the 26% training deficit (n=109) and 24% IT support issue (n=98) showed infrastructure limitations [11,15], hindering sustainability as noted in a systematic review [16].

Limitations of the study

The study's single-center design limits generalizability to other Nigerian or LMIC settings. The cross-sectional nature precludes causality, and self-reported data may introduce bias. The 415 sample (post-data cleaning) may not fully represent EMR user diversity.

Recommendations

Routine training on EMR should be conducted for all clinical staff to enhance satisfaction and usability. Improvements in IT support should be instituted to reduce technical disruptions and ensure system reliability. User-centered interface redesign should be done to streamline documentation and navigation. Regular system audits should be done to sustain service delivery improvements.

## Conclusions

Healthcare professionals at FMC Gusau perceive EMRs as improving healthcare service delivery by 67.2-80.4% in documentation and record access. However, satisfaction remains moderate, highlighting the need to address training and technical barriers.

## Appendices

Questionnaire: Healthcare professionals' satisfaction and influence of Electronic Medical Record on healthcare services in Federal Medical Center, Gusau

Section 1: Demographics

1. What is your professional role?

Medical Doctor

Nurse

Pharmacist

Other (please specify): _________

2. Age: _________ years

3 . Gender:

I. Male     II. Female

4 . Have you received training on the EMR system?

I. Yes      II. No

5. How long have you been working at FMC Gusau?

(Specify in years): _________

Section 2: Healthcare Providers’ Satisfaction with EMR Systems

6. How easy is it to navigate the EMR system?

I. Very easy II.  Somewhat easy III. Neutral  IV. Somewhat difficult  V. Very difficult

7. How satisfied are you with the overall user-friendliness of the EMR system?

I. Very satisfied     II. Somewhat satisfied    III. Neutral    IV. Somewhat dissatisfied

V. Very dissatisfied

8. How satisfied are you with the timeliness of the EMR system in retrieving information?

I. Very satisfied      II. Somewhat satisfied      III. Neutral    IV. Somewhat dissatisfied

V. Very dissatisfied

9. How often do you experience technical issues with the EMR system?

I. Never     II. Rarely     III. Occasionally     IV. Frequently    V. Almost always

10.How would you rate your overall satisfaction with the EMR system?

I. Excellent                 II. Good                 III. Fair            IV. Poor           V. Very poor

Section 3: Clinical Workflow Efficiency

11. How well does the EMR system streamline documentation processes?

I. Very well        II. Somewhat well      III. Neutral    IV. Poorly     V. Very poorly

12.How easy is it to retrieve patient records using the EMR system?

I. Very easy           II. Somewhat easy        III. Neutral           IV. Somewhat difficult   V. Very difficult

13.How has the EMR system impacted time spent on patient documentation?

I. Significantly reduced      II.Slightly reduced   III.  Neutral       IV. Slightly increased

V.   Significantly increased

14.How would you rate the EMR system’s ability to improve interdepartmental communication?

I. Excellent                   II.Good                 III.Fair             IV. Poor         V.Very poor

15.How well does the EMR system help reduce delays in accessing patient information?

I. Very well           II. Somewhat well       III. Neutral        IV. Poorly     V.Very poorly

Section 4: Resource Allocation

16.How well does the EMR system optimize resource use within the hospital?

I. Very well       II. Somewhat well     III. Neutral    IV. Poorly     V. Very poorly

17.How effective is EMR system in managing staff workload?

I. Very effective  II. Somewhat effective   III. Neutral  IV. Somewhat ineffective

V. Very ineffective

18.How well does the EMR system support financial resource tracking?

I. Very well         II. Somewhat well     III. Neutral         IV. Poor       V. Very poor

19.How confident are you in the EMR system’s ability to enhance technological investments?

I. Very confident    II. Somewhat confident    III. Neutral   IV. Somewhat not confident     V. Not confident at all

20.How would you rate the overall impact of the EMR system on hospital resource allocation?

I. Excellent             II. Good            III.   Fair              IV.  Poor           V.  Very poor

Section 5: Challenges of EMR Implementation

21.What are the primary challenges you face with the EMR system? (Select all that apply):

I. Lack of training   II. Poor design   III. Downtime issues  IV.  Limited IT support

V.  Other (please specify): _________

22.How frequently do EMR-related issues disrupt your workflow?

I. Always             II.  Often             III.  Sometimes       IV.  Rarely     V.   Never

23.How confident are you in the hospital’s ability to address EMR-related challenges?

I. Very confident     II. Somewhat confident    III. Neutral    IV. Somewhat not confident        V. Not confident at all

24.How would you rate the security measures of the EMR system in protecting patient information?

I.    Excellent          II. Good        III.  Fair        IV. Poor          V.  Very poor

Thank you for participating in this study
